# Impact of hypoglycemic episodes on health-related quality of life of type-2 diabetes mellitus patients: development and validation of a specific QoLHYPO^©^ questionnaire

**DOI:** 10.1186/s12955-018-0875-1

**Published:** 2018-03-23

**Authors:** Domingo Orozco-Beltrán, Sara Artola, Margarida Jansà, Martin Lopez de la Torre-Casares, Eva Fuster

**Affiliations:** 1 Cabo Huertas Healthcare Center, San Juan de Alicante, Universidad Miguel Hernández, Alicante, Spain; 2 José Marvá Healthcare Center, RedGDPS Foundation, Madrid, Spain; 3 Endocrinology and Diabetes Center, Hospital Clínic, Barcelona, Spain; 4 Hospital Virgen de las Nieves, Granada, Spain; 5 Novartis, Barcelona, Spain

**Keywords:** Health-related quality of life, Type-2 diabetes mellitus, Hypoglycemia, Questionnaire development, Questionnaire validation

## Abstract

**Background:**

Hypoglycemia is a limiting factor to achieving optimal glycemic control in patients with type-2 diabetes mellitus (T2DM), increasing risk of death and complications, reducing health-related quality of life (HRQoL) and work productivity and increasing healthcare costs.

The study’s primary objective was to develop and validate a specific questionnaire to assess the impact of hypoglycemia on the HRQoL of T2DM patients (QoLHYPO^©^ questionnaire).

**Methods:**

A two-phase multicenter prospective, longitudinal, observational, epidemiologic study of consecutively enrolled patients, not involving any drug, was conducted: In phase 1 (questionnaire development), patients who had given their written informed consent, who were at least 30 years of age, had been diagnosed with T2DM at least 5 years prior, had an HbA1c test in the previous 3 months, and a hypoglycemic episode in the previous 6 months were included. To validate the questionnaire and assess reliability and responsiveness, phase 2 included two cohorts of patients. Patients in the reliability cohort would likely have stable clinical course during the 3 weeks following inclusion in the study and patients in the responsiveness cohort would likely experience changes in their clinical course in the 3 months after enrollment.

**Results:**

Phase 1 included 168 patients: 10 attended semi-structured interviews, 18 for face validity, and 140 for the pilot test (Rasch analysis). Phase 2 included 227 patients: 142 in the reliability cohort and 85 in the responsiveness cohort.

Of the 37 items initially included in Phase 1, 11 (floor/ceiling effect analysis) and 13 (Rasch analysis) were discarded. The final version of the questionnaire consisted of 13 items.

Phase 2 results showed the questionnaire was unidimensional and able to accurately assess HRQoL. Intra-observer reproducibility (ICC = 0.920) and internal consistency (Cronbach’s alpha: visit 1 = 0.912; visit 2 = 0.901) were high, showing high reliability. Internal responsiveness was moderate (standardized effect size 0.5-0.8) and external responsiveness was lower (AUC > 0.5; not statistically significant). Minimal clinically important difference (MCID) was estimated to be 3.2 points.

**Conclusions:**

The QoLHYPO^©^ questionnaire is a tool that can be used in routine clinical practice to assess the impact of hypoglycemia on the HRQoL of T2DM patients.

**Electronic supplementary material:**

The online version of this article (10.1186/s12955-018-0875-1) contains supplementary material, which is available to authorized users.

## Background

Diabetes mellitus (DM), especially type-2 DM (T2DM), is one of the most common chronic diseases, with rising global prevalence. In 2014 the International Diabetes Federation (IDF) estimated that worldwide, 8.8% of adults between the ages of 20 and 79 years (415 million persons) were living with diabetes and that it caused 4.9 million deaths each year [1]. The same authors estimate that by 2040 the number of diabetic persons across the world will rise to 642 million [1]. In Spain, the Di@bet.es study showed 13.8% T2DM prevalence (6% unknown), reaching 20% in people aged 60-75 years [2].

Despite improvements made towards the achievement of optimal control, there are still large numbers of patients who do not reach this goal and, therefore, are at risk of developing complications [3]. Thus, the estimated life expectancy for a 40-year-old patient with T2DM is shortened by 6 to 7 years [4].

Since fear of hypoglycemia may contribute to a lack of adherence to treatment, hypoglycemia is one of the main limiting factors for controlling glycaemia in T2DM patients [5, 6]. Hypoglycemia, which is defined as blood glucose concentration ≤ 70 mg/dL [7], is typically characterized by its sudden onset and by physical and psychological symptoms such as shakiness, sweating, drowsiness, nausea, poor motor coordination, mental confusion, irritability and loss of consciousness [5]. Sometimes it presents with non-specific or atypical symptoms and may even be asymptomatic, making it even more difficult to detect, assess and treat.

The prevalence of hypoglycemic episodes in patients with T2DM varies according to the treatment received, with patients who are treated with insulin at highest risk of experiencing them [8]. Moreover, it is important to note that nearly half of T2DM patients rarely or never informed their doctor about non-severe hypoglycemia [9, 10].

Hypoglycemia is also associated with the development of long-term complications and diminished work productivity [11] and ability to perform daily activities [9, 12]. In comparison with patients who have never experienced hypoglycemia, patients who experienced hypoglycemia tend to have a higher rate of absenteeism (7.6% vs. 4.4; *p* < 0.05), greater shortcomings while at work or presentism (21.3% vs. 15.1%; *p* < 0.05) and report greater interference with the performance of daily activities (19% vs. 9.3%; *p* < 0.05) [9]. The economic impact of hypoglycemia on the use of healthcare resources is significant, as it leads to higher healthcare costs for these patients, primarily due to an increase in hospitalizations, emergency room visits or specialized healthcare [10, 13–16]. In Spain, the cost per episode of severe hypoglycemia is estimated at €3500, without taking into account the impact of hypoglycemia on patients’ work productivity [12].

Several studies have highlighted the negative impact of hypoglycemia on patients’ health-related quality of life (HRQoL) [9, 11, 17–19]. Assessment of the HRQoL of patients with hypoglycemia using generic health questionnaires such as Short Form (SF)-36, SF-12 or EuroQoL-5D (EQ-5D) has shown the impairment of both physical and mental components of HRQoL [9, 11, 16]. Phase III clinical trials have also demonstrated the association between hypoglycemia and HRQoL, with worse HRQoL reported for patients who experience hypoglycemic episodes more frequently [19]. The use of diabetes-specific HRQoL questionnaires such as the Audit of Diabetes-Dependent Quality of Life (ADDQoL) in patients with and without hypoglycemia revealed the negative impact on the HRQoL of patients who experience hypoglycemia, however no significant differences in the general items were observed between patients with hypoglycemic events and patients without [18]. This could be explained by the fact that this questionnaire is less sensitive than a more specific one [18] and highlighted the need to developed a specific questionnaire able to assess the impact of hypoglycemia on patients’ HRQoL.

Since hypoglycemia is a major barrier to achieving the glycemic goal in T2DM patients, and the impact on HRQOL and worry they cause can lead to the patient developing self-regulation attitudes towards medication and low adherence to it to avoid them, incorporating the examination of the impact of hypoglycemia on HRQoL can contribute to improve the management of T2DM patients and promote a therapeutic approach directed toward the patients’ needs, contributing to a better perception of HRQoL. Besides some T2DM specifics HRQoL questionnaires have been developed, at the time of study development, a specific questionnaire to assess this impact was not available. For this reason, the main purpose of this study was to develop and validate a HRQoL questionnaire to assess the impact of hypoglycemic episodes on T2DM patients’ HRQoL.

## Methods

### Design and scope of the study

**Fig. 1:**
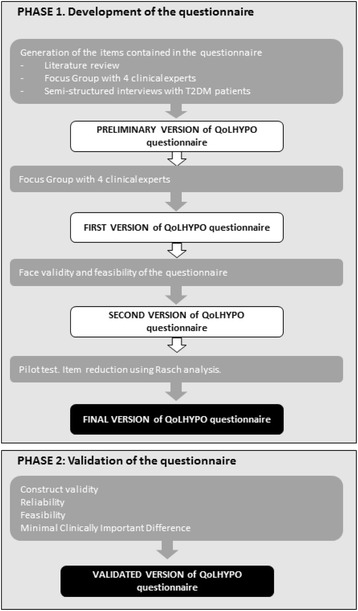
Study flow chart

In order to develop and validate the QoLHYPO^©^ questionnaire, a multicenter, longitudinal, observational, epidemiologic study with consecutive enrollment of patients, not involving any drug, was designed. The study was divided into two phases (Fig. 1): Phase 1, development of the questionnaire (between September 2013 and July 2014); and Phase 2, validation of the questionnaire (from October 2014 to July 2015).

The study was conducted at Primary Healthcare centers and Endocrinology Departments within the Spanish healthcare system.

The study was classified as a non-post-authorization observational study by the AEMPS - *Agencia Española de Medicamentos y Productos Sanitarios* (Spanish Agency for Medicines and Medical Devices) and it was approved by the Ethics Committee of Hospital Clínic of Barcelona (25 July 2013).

All T2DM patients included in the study gave their written consent to participate in the study.

### Phase 1: Development of the questionnaire

#### Generation of the items contained in the questionnaire

First, a review of the literature was conducted to identify the domains and descriptors of HRQoL in T2DM patients who experienced hypoglycemia. Subsequently, a focus group with four clinical experts evaluated the domains and descriptors found in the review and provided any additional information that may not had been identified in the literature [20].

Based on the conclusions of the focus group, a script was designed for conducting semi-structured interviews with patients. The aim of these interviews was to find out the patients’ perceptions regarding the HRQoL domains and to draw up an initial list of the items for inclusion in the preliminary version of the questionnaire [21]. Patients 30 years of age or older who had been diagnosed with T2DM for at least 5 years, and who had had an HbA1c test in the previous 3 months and a hypoglycemic episode in the 6 months prior to the study were interviewed.

Finally, the preliminary version of the questionnaire was reviewed and validated by the same experts who participated in the first focus group. The first version of the questionnaire resulted from this discussion.

#### Face validity and feasibility of the questionnaire

In order to determine the ease with which patients answered the first version of QoLHYPO^©^, face validity and feasibility were assessed. A pilot test with T2DM patients was performed to evaluate the clarity of the wording of the items, their importance and the perceived burden (difficulty and time spent on the questionnaire), using a 5-point Likert scale (0 = not at all satisfactory to 5 = very satisfactory). The face validity and feasibility test population included adult patients ≥30 years old, diagnosed with T2DM for at least 5 years, with an HbA1c test in the previous 3 months and who had a hypoglycemic episode in the 6 months prior to the study.

Following this assessment, items that had scored equal to or greater than 4 by at least 75% of patients were selected for inclusion in the second version of QoLHYPO^©^ questionnaire.

Statistical analysis was performed using SPSS Statistics v.20 software.

#### Pilot test. Item reduction using Rasch analysis

To reduce the number of items, floor and ceiling effects and Rasch analysis were applied. Floor and ceiling effects were determined if at least 35% of patients had used the lowest (floor) or highest (ceiling) response categories. Items that presented with floor and/or ceiling effects were removed from the questionnaire. It does not exist a cut-off value to define floor and ceiling effects, it usually is an arbitrary value which depends on the study population [22]. Since the study sample is composed by a high proportion of patients (58%) with mild hypoglycaemia episodes, in order to be more sensitive to the severity of hypoglycaemias a cut-off value of 35% was used. Rasch analysis is a mathematical modeling technique that is widely used in the development of questionnaires [23–27]. The main characteristic of the model is that item and person parameters can be estimated independently of the characteristics of the sample and the difficulties of the items, respectively [28]. Rasch analysis estimates the thresholds or degrees of difficulty of the response categories for each item. These thresholds are displayed graphically by means of characteristic curves, which must appear in the same ascending order as the category responses (the higher the score, the better the HRQoL). These graphics allow identifying redundant response categories where the curves of different categories overlap [29]. The presence of redundant categories indicates an excess of response categories, so they were recoded and merged. Items that still displayed overlapping or unordered curves after recoding were removed from the questionnaire.

After assessment of the characteristic curves, the fit of the items to the Rasch model was analyzed through the infit and outfit statistics calculation. Both statistics are derived from the squared standardized residuals, with a goodness of fit range between 0.5 and 1.5 in polytomous responses [30, 31]. Items whose infit and outfit values exceeded the established range were eliminated from the questionnaire so that the final version of the QoLHYPO^©^ was constituted by the remaining ones.

A total of 150 T2DM patients comprise the sample. Sample size estimation was based on the minimum number required for Rasch parameter estimation with a stability of 0.5 logits and 99% confidence [32]. An additional 7% of patients were recruited for possible losses. Patients had to meet the following criteria: be at least 30 years old, have been diagnosed with T2DM for a minimum of 5 years, have had at least one HbA1c test in the 3 months prior to the study, have presented at least one hypoglycemic episode in the 6 months prior to the study, and have given their consent to participate in the study.

The R package eRm was used to perform Rasch analysis [33, 34].

### Phase 2: Validation of the questionnaire

During Phase 2 of the study, the questionnaire that had been developed in Phase 1 was validated. Two cohorts of specific patients were included so as to assess reliability and responsiveness, respectively. Patients in both cohorts attended two visits: at baseline and 3 weeks later, in the case of the reliability cohort, or 3 months later, in the case of the responsiveness cohort. In addition to the inclusion criteria established for Phase 1 of the study, a specific additional criterion was defined for each cohort. Patients in the reliability cohort were expected to have a stable clinical course during the 3 weeks following their inclusion in the study. Patients in the responsiveness cohort were expected to have an unstable clinical course during the 3 months following their inclusion in the study, as determined by greater or lesser incidence and/or changes in the severity of hypoglycemic episodes.

The patients completed the QoLHYPO^©^, ADDQoL-19 and EQ-5D-3 L questionnaires at each visit. To measure reliability, a sample size composed of 154 patients with a 95% confidence level, 95% power and 0.3 effect size was estimated. The same parameters were used in the responsiveness cohort but with an effect size of 0.4 (it was expected to obtain greater differences between visits), so a sample size of 84 patients was estimated. G*Power software was used to calculate sample size [35].

#### Construct validity

To analyze the construct validity of the QoLHYPO^©^ questionnaire, the responses to the questionnaire obtained from both cohorts at the baseline visit were used. For the assessment of the number of domains that constitute the QoLHYPO^©^ questionnaire, several exploratory factor analysis techniques were used (*optimal coordinate*, *acceleration factor, Velicer’s minimum average partial test* - original and modified, *Horn’s parallel analysis*) [36]. The basis of all these techniques is that every factor (or domain) is defined by an eigenvalue, so that the greater the eigenvalue, the greater the variance explained by the factor. These analyses were performed with the R package *paramap* statistical program [33, 37]. In addition, to evaluate whether the QoLHYPO^©^ questionnaire was measuring the same characteristics as specific questionnaire ADDQoL-19 and generic questionnaire EQ-5D-3 L, the multitrait-multimethod matrix was calculated using Spearman’s rho correlations between the scores of the various questionnaires.

#### Reliability

The reliability of a questionnaire refers to the degree of consistency and to intra-observer reproducibility, i.e., the equivalence between repeated measurements in the same individual. It was evaluated using test-retest reliability and internal consistency based on the responses given to the QoLHYPO^©^ questionnaire by patients included in the reliability cohort during the baseline and follow-up visits [38–40]. Internal consistency was measured using Cronbach’s alpha on the QoLHYPO^©^ total score. Test-retest reliability was assessed using the intra-class correlation coefficient (ICC) for the total score and Cohen’s Kappa coefficient for the responses to each item. Cronbach’s alpha values above 0.70 were regarded as satisfactory internal consistency [41]. ICC and Kappa values above 0.75 and 0.70, respectively, were regarded as indicators of good reliability [40].

SPSS v.20 software was used to compute Cronbach’s alpha and ICCs, and Cohen’s Kappa coefficient was computed using VassarStats software [42].

#### Responsiveness

Responsiveness was measured based on the responses to the QoLHYPO^©^ questionnaire given by the patients, who constituted the responsiveness cohort. In the follow-up visit (at 3 months), patients had to report how their health status had changed with respect to the baseline visit. They responded to an anchor question to assess the evolution in their health status from the previous visit in relation with the T2DM, and therefore, with the hypoglycemic episodes. Answers were scored on a 5-point Likert scale: 1 = much worse, 2 = worse, 3 = no change, 4 = better and 5 = much better.

Responsiveness can be internal, referring to the ability of a measure to change over a predefined time period, or external, referring to the degree to which change in a measure relates to a change in a reference measure of clinical or health status [43]. It is important to notice that the objective of the responsiveness assessment was the ability of the questionnaire to detect changes, no the direction of the changes. Internal responsiveness was measured with standardized response mean (SRM) [44]:

$$ SRM=\frac{mean\ {\left({QoLHYPO}_{visit1}-{QoLHYPO}_{visit2}\right)}_{improved}}{SD{\left({QoLHYPO}_{visit1}-{QoLHYPO}_{visit2}\right)}_{stable}} $$

assuming that the group that had experienced improvement had responded to the anchor question with “better” or “much better” while those who had remained stable had responded “no change”. Values less than 0.2, between 0.2 and 0.5, between 0.5 and 0.8, and greater than 0.8 indicated slight, low, moderate and high sensitivity, respectively [43, 45]. External responsiveness was based on Receiver Operating Characteristics curves (ROC) [43, 46]. To assess external responsiveness, patients were coded as experiencing improvement (responded with “better” or “much better”) or no improvement (responded with “much worse”, “worse” or “no change”).

#### Minimal clinically important difference

Minimal clinically important difference (MCID), defined as the smallest change in score in the questionnaire that could be considered significant, was based on the above-mentioned anchor questions, which allowed selecting patients who had experienced an improvement (“better”) [47]. Similarly, the professionals who recruited the patients also responded to an equivalent anchor question to assess their patients’ clinical course. The difference in score in the anchor question of the QoLHYPO^©^ questionnaire between the baseline visit and follow-up visit of patients whose physician and they themselves reported an improvement was used to determine MCID.

## Results

### Phase 1. Development of the questionnaire

#### Generation of the items contained in the questionnaire

After reviewing the literature and holding the two focus groups with 4 T2DM management experts and 10 interviews with T2DM patients, the first version of the QoLHYPO^©^ questionnaire was developed. Table 1 shows the items included in the first version of the QoLHYPO^©^ questionnaire.

**Table 1:** Items included in version 1 of the QoLHYPO^©^ questionnaire

When my blood sugar drops…	
Social relationships	5.1. I don’t feel like talking to anyone
	5.2. I can meet with my friends without waiting to recover
	5.3. I can have sex
	5.4. My family and friends understand what is happening to me
	5.5. I feel supported and aided by my family
Mood	5.6. I have the feeling that what my healthcare provider is telling me to do does not help to control a low blood sugar
	5.7. I feel down, because in spite of following the advice of my healthcare provider, my blood sugar level has dropped
	5.8. I get moody
Sleep	5.9. I wake up in the middle of the night and I have trouble sleeping the next few nights
Changes in daily activity	5.10. I can carry on with my regular activity for the rest of the day
	5.11. If I’m driving and I get dizzy, I have to stop immediately
	5.12. I have a hard time doing housework
	5.13. I can run to catch a bus or to cross the street
Blood sugar control	5.14. I check my blood sugar more often to be on the safe side until it is under control
	5.15. I don’t mind having to make changes in my medication
Physical condition	5.16. I feel so tired I don’t feel like doing anything
Think of each of the following statements in relation to your drops in blood sugar.	
Social relationships	6.1. The low blood sugar affects the relationship with my family.
Relationship with healthcare provider	6.2. A good relationship with the healthcare provider makes me feel more secure and I am less worried
	6.3. A good relationship with the healthcare provider is important to me to address the concerns I have about my drops in blood sugar
	6.4. Telling my healthcare provider the truth is fundamental so she/he can help me manage my blood sugar drops
Mood	6.5. Because of my blood sugar drops, I’ve lost self-confidence
	6.6. I’m afraid of being alone and fainting
	6.7. Generally speaking, I worry more about my blood sugar dropping
	6.8. When I go to bed I’m afraid my blood sugar will drop while I’m asleep
Changes in daily activity	6.9. When I’m job hunting, it’s better not to talk about my drops in blood sugar
	6.10. Holding on to my job may be hard if I have continuous drops in my blood sugar
	6.11. The drops in my blood sugar prevent me from performing my job normally
	6.12. I’m worried I won’t be able to get a driver’s license because of my drops in blood sugar
	6.13. I don’t drive because I’m afraid of something happening if my blood sugar drops while I’m driving
Physical condition	6.14. I exercise in spite of the drops in blood sugar
	6.15. My blood sugar drops quickly when I run so I always have to carry food with me
	6.16. I exercise less than I should because I’m afraid of my blood sugar dropping
	6.17. If I’m going to exercise more, I eat more to prevent my blood sugar from dropping
Blood sugar control	6.18. Because of my drops in blood sugar, I need to go to the emergency room more often
	6.19. My drops in blood sugar have made me more aware about what I eat and the activities I perform
	6.20. It’s annoying to have to eat when I’m not hungry in order to avoid a drop in blood sugar
	6.21. Having to check my blood sugar levels when I notice the symptoms that it is dropping helps me to take appropriate steps to control it

#### Face validity and feasibility of the questionnaire

A total of 18 T2DM patients participated in the face validity and feasibility of the questionnaire. Related to questionnaire format, approximately 81 and 94% of participants scored 4 and 5 (from 1 = not at all satisfactory, to 5 = very satisfactory), respectively, indicating that they were satisfied with it. The mean time required to complete the questionnaire was 13 min (SD: 5 min., range 7-25 min).

After questionnaire face validity and feasibility, the second version of the questionnaire was developed. It included four general items about the impact of hypoglycemia on patients’ HRQoL (frequency and severity of hypoglycemia, knowledge to control hypoglycemia and frequency of use of glucometer) and two sets of questions with 16 and 21 items, respectively. First set of questions was related to the patients’ feelings when their blood sugar drops. The second one, explores patients’ perceptions in relation to their drops in blood sugar. Each set of questions had five categories of response (0 = Never, 1 = Rarely, 2 = Sometimes, 3 = Often, 4 = Always).

#### Pilot test. Item reduction using Rasch analysis

A total of 160 patients with T2DM from 12 Spanish autonomous regions were included in the pilot test. Of these patients, 20 did not meet the inclusion criteria so the final sample consisted of 140 T2DM patients. Table 2 shows the main characteristics of the study population.

**Table 2:** Clinical and sociodemographic characteristics of the patients included in the study (Phase 1 and Phase 2)

Patient characteristics	Phase 1 (*n* = 140)	Phase 2 (*n* = 227)
Age (mean years, SD)	63.0 (9.9),	62.7 (11.0)
Gender (% men)	55.0	54.6
Marital status (% married)	70.7	74.00
Level of education (%)		
*No schooling*	10.7	7.9
*Primary*	43.6	41.4
*Secondary*	19.3	30.4
*Vocational*	12.9	7.9
*University and post-graduate*	13.6	12.4
Employment status (%)		
*Active*	33.6	34.4
*Unfit for work*	0.7	1.3
*Unemployed*	7.9	3.5
*Retired*	37.9	44.5
*Homemaker*	20.0	15.0
*Other*	0.0	1.3
BMI (Kg/m2, SD)	29.3 (5.0)	29.1 (4.6)
Sedentariness (%)	25.0	19.0
Time since T2DM diagnosis (years, SD)	14.6 (6.9)	12.6 (7.4)
Family history of T2DM (%)	58.6	63.4
HbA1c (mean, min-max)	7.4 (5.3-10.8)	7.4 (4.6-17.1)
Presence of T2DM-related complications (%)	37.1	35.7
*Microvascular (% of patients with complications)*	76.9	56.8
*Macrovascular (% of patients with complications)*	48.1	80.2
Charlson index (mean, SD)	2.2 (1.7)	1.9 (1.8)
Number of hypoglycemic episodes in the last 6 months (mean, SD)	5.5 (11.1)	5.1 (7.1)
Number of hypoglycemic episodes in the last 6 months confirmed with glucose meter (mean, SD)	3.5 (3.5)	3.5 (5.3)

Table 3 shows the answers to the 4 general items about the hypoglycemia in each phase of the study. As in Phase 1 a high proportion of patients had mild to very mild hypoglycemia (57.67%), a high cut-off value (35%) was used to assess floor and ceiling effects in order to make the questionnaire more sensitive to the severity of the hypoglycemia. After floor and ceiling effects testing (Table 4), 11 items were discarded. The first Rasch analysis was therefore conducted on the remaining 26 items. To estimate the threshold of HRQoL of the response categories for each item, a graphical representation of characteristic curves for each item was used. It allowed assessing the scale scoring system through a graphic display of the probability of patients answering each response category for each item, according to the individual’s ability (ability = HRQoL). Each item provided five response options, where 0 was the least favorable case (worse HRQoL) and four was the most favorable (better HRQoL).

**Table 3:** Distribution of responses to general items about hypoblycemia

General items about hypoglycemia	Phase 1 (*n* = 140)	Phase 2 (*n* = 227)
Frequency of hypoglycemia (%)		
*Hardly ever*	32.35	29.1
*Sometimes*	57.35	56.8
*Frequently*	10.29	14.1
Severity of hypoglycemia (%)		
*Very mild*	20.44	21.2
*Mild*	37.23	34.5
*Moderate*	32.12	35.2
*Severe*	10.22	8.4
*Very severe*	0	0.4
Knowledge to control hypoglycemia (%)		
*Without knowledge*	7.30	7.5
*Need for more knowledge*	49.64	44.9
*Good knowledge*	38.69	40.1
*Very good knowledge*	4.38	7.5
Frequency of use of glucometer (%)		
*Never*	4.38	10.6
*Hardly ever*	11.68	11.5
*Sometimes*	34.31	30.0
*Frequently*	34.31	21.1
*Always*	15.33	26.9

**Table 4:** Distribution of responses to assess floor and ceiling effects

Item	Never (%)	Rarely (%)	Sometimes (%)	Often (%)	Always (%)
5.1	13.14	17.52	37.96	20.44	10.95
5.2	22.63	21.17	27.74	16.79	11.68
5.3	38.81 (floor effect)	23.88	15.67	11.19	10.45
5.4	4.44	7.41	21.48	29.63	37.04 (ceiling effect)
5.5	1.46	4.38	13.87	18.98	61.31 (ceiling effect)
5.6	31.39	29.20	21.17	8.03	10.22
5.7	24.09	24.09	35.77	10.95	5.11
5.8	15.33	18.25	35.77	21.90	8.76
5.9	24.82	32.12	29.93	8.03	5.11
5.10	3.65	8.76	18.98	45.26	23.36
5.11	23.48	18.94	15.15	12.88	29.55
5.12	16.06	18.98	37.23	16.06	11.68
5.13	22.63	27.01	22.63	16.79	10.95
5.14	9.49	17.52	29.20	27.74	16.06
5.15	23.36	24.82	24.82	16.06	10.95
5.16	13.14	17.52	39.42	19.71	10.22
6.1	31.39	24.09	30.66	9.49	4.38
6.2	1.46	0.73	12.41	35.04	50.36 (ceiling effect)
6.3	1.46	0.73	8.76	26.28	62.77 (ceiling effect)
6.4	0	0.74	7.41	14.81	77.04 (ceiling effect)
6.5	24.26	25.00	38.24	6.62	5.88
6.6	18.25	25.55	29.20	16.06	10.95
6.7	8.76	26.28	37.96	17.52	9.49
6.8	18.98	28.47	32.12	9.49	10.95
6.9	37.40 (floor effect)	15.27	18.32	18.32	10.69
6.10	39.39 (floor effect)	17.42	16.67	17.42	9.09
6.11	33.09	18.38	31.62	6.62	10.29
6.12	47.73 (floor effect)	16.67	21.97	4.55	9.09
6.13	46.21 (floor effect)	25.00	18.18	5.30	5.3
6.14	16.79	19.71	33.58	18.25	11.68
6.15	20	23.70	26.67	16.30	13.33
6.16	24.09	27.74	32.85	10.22	5.11
6.17	19.26	22.96	33.33	13.33	11.11
6.18	41.61 (floor effect)	28.47	23.36	5.11	1.46
6.19	12.5	14.71	22.79	30.15	19.85
6.20	21.9	26.28	35.04	10.95	5.84
6.21	9.49	9.49	21.90	24.82	34.31

**Fig. 2:**
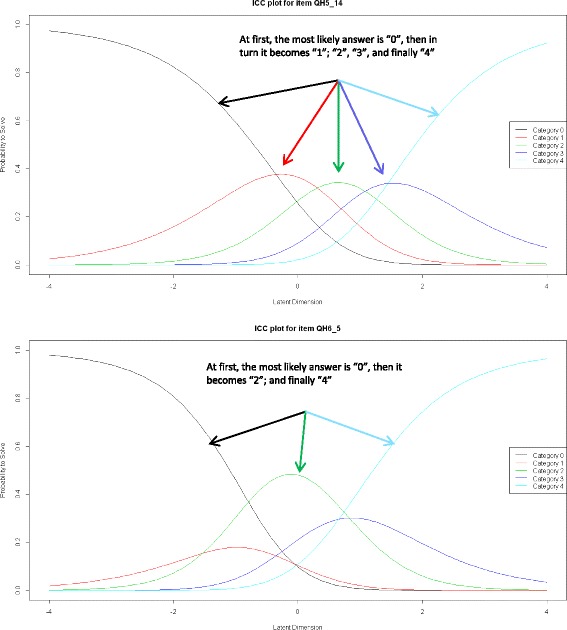
Characteristic curves for items 5.14 and 6.5

For most of the items, the probabilities of response overlapped, suggesting that the patients were not using the full range of options allowed by the scoring system, which implied that the categories were redundant (see Additional file 1). These results showed the need to recode the responses by merging the categories: “Never” with “Rarely” and “Often” with “Always”, so that only three response categories remained. Figure 2 shows the characteristic curves for items 5.14 (not overlapping) and 6.5. (overlapping).

After recoding the response categories, a second Rasch analysis was conducted. New characteristic curves continued showing overlapping in 11 items (see Additional file 2). They were therefore removed from the questionnaire. Rasch analysis was again performed on the remaining set of 15 items (26-11 = 15) with three ordered response categories to assess proper fit of the items to the Rasch model. Two items had infit and outfit values that exceeded the 0.5-1.5 range and were therefore discarded. Finally, another Rasch analysis was conducted on the resulting set of 13 items, which determined the goodness of fit of the items for measuring the construct of interest (Table 5). These items constituted the final version of the QoLHYPO^©^ questionnaire (see Additional file 3).

**Table 5:** Infit and outfit statistics after second adjustment

Item	Outfit	Infit
5.1	1.048	0.999
5.7	0.933	0.987
5.8	0.834	0.894
5.9	1.039	1.050
5.12	0.711	0.742
5.16	0.774	0.814
6.1	0.673	0.750
6.5	0.863	0.898
6.7	0.985	0.975
6.8	1.155	1.014
6.11	1.127	1.076
6.16	1.221	1.051
6.20	1.274	1.177

### Phase 2. Validation of the questionnaire

A total of 254 patients with T2DM participated in Phase 2 of the study. As 27 of them did not meet the selection criteria, they were excluded. The final sample consisted of a total of 227 T2DM patients residing in 12 autonomous regions, 142 from the reliability cohort and 85 from the responsiveness cohort (Table 2).

#### Construct validity

The QoLHYPO^©^ questionnaire responses reported by the participants included in Phase 2 (*n* = 227) were used for the construct validity analysis.

All the techniques used for the exploratory factor analysis showed that the QoLHYPO^©^ questionnaire consisted of a single factor and was therefore unidimensional. The multitrait-multimethod matrix indicated that there was a significant correlation (rho = 0.557; *p* < 0.001) between the QoLHYPO^©^ and the DM-specific questionnaire ADDQoL-19. A significant correlation was likewise observed between the dimensions of the generic HRQoL questionnaire, EQ-5D-3 L, and the scores obtained in the QoLHYPO^©^ questionnaire [mobility rho = − 0.216 (*p* = 0.001), self-care rho = − 0.259 (*p* < 0.01), usual activities rho = − 0.032 (*p* < 0.01), pain/discomfort rho = 0.265 (*p* < 0.01), anxiety/depression rho = − 0.346 (*p* < 0.01), VAS rho = − 0.446 (*p* < 0.01)].

#### Reliability

Reliability analysis was conducted based on the results reported by the reliability cohort (*n* = 142 patients).

The internal consistency of the QoLHYPO^©^, which was measured using Cronbach’s alpha, was high at both visits (visit 1 = 0.912; visit 2 = 0.901). The values obtained as a result of test-retest showed that the QoLHYPO^©^ has good reliability [ICC = 0.920 (CI95%: 0.890-0.942); and kappa> 0.60 for all items].

#### Responsiveness

For the analysis of responsiveness we used the results obtained in the responsiveness cohort (*n* = 85 patients).

Internal responsiveness was determined based on patients’ self-reported health improvement. The results showed a standardized effect size of 0.676, indicating moderate internal responsiveness (between 0.5 and 0.8). External responsiveness assessment using ROC curve analysis did not yield significant results.

#### Minimal clinically important difference (MCID)

Analysis of MCID showed that the QoLHYPO^©^ questionnaire was able to detect improvement in health when there was a 3.2 point increase between visits.

### Impact of hypoglycemia on patients’ HRQoL

Assessment of T2DM patients’ HRQoL using the three questionnaires included in the study (QoLHYPO^©^, ADDQoL and EQ-5D-3 L), showed that the HRQoL of the T2DM patients included in Phase 2 of the study was compromised (Table 6).

**Table 6:** Health-related quality of life of patients included in Phase 2 of the study

Measurement instrument (scoring scale)	Score obtained (SD)
QoLHYPO (0 = worst possible HRQoL; 26 = best possible HRQoL)	15.9 (SD: 6.7)
ADDQoL (−9 = maximum negative impact to 3 = maximum positive impact)	−2.0 (SD: 1.7)
VAS EQ-5D-3 L (0 = worst state of health and 100 = best state of health)	65.7 (SD: 16.3).

The analysis of the results obtained from the QoLHYPO^©^ questionnaire showed that aspects such as social relationships (talking to people) or mood (becoming irritable) were most affected by hypoglycemia (> 25% of patients indicated that this aspect was always affected by hypoglycemic episodes).

Determining HRQoL using a generic HRQoL questionnaire such as EQ-5D-3 L confirmed this impairment, establishing that 29.2% of patients had difficulty walking, 15.0% had problems with self-care, 26.0% reported having difficulties performing their usual activities, 46.7% suffered pain or discomfort, and 33.0% felt anxiety or depression.

## Discussion

Hypoglycemia and fear of hypoglycemia is a major problem for patients with diabetes, posing a barrier to good glycemic control [48, 49]. The negative consequences of hypoglycemia for both the daily life and well-being of the patient, confirm the importance to include, in the routine clinical practice, strategies of identifying patients with a high impact on HRQoL due to these episodes. However, a recent study conducted in 661 primary and specialized care centers belonging to Spain’s public healthcare system has shown that doctors do not use questionnaires that measure patient HRQoL in routine clinical practice [18].

Currently, some generic HRQoL questionnaires are available, such as EQ-5D [50] or SF-36 [51], that may be useful for determining T2DM patients’ HRQoL [52]. Although the use of these questionnaires allows comparing the HRQoL of T2DM patients with the HRQoL of patients with other diseases or the HRQoL of the general population, they are not sensitive to changes associated with the specific symptoms or characteristics of a disease. On the other hand, specific questionnaires for determining the impact of diabetes on patients’ HRQoL [53, 54], for measuring fear of hypoglycemia [55], for assessing the attitude that patients with diabetes have with regard to hypoglycemia [56] or for determining the frequency with which it occurs [57] are also available. However, none of them has been validated in a Spanish population and none determines the impact of hypoglycemia on HRQoL. Hence, the information obtained by using a questionnaire that allows determining the impact hypoglycemia has on the HRQoL of T2DM patients could contribute to identifying those patients who may be at greatest risk and, consequently, help clinician to promote strategies to a target population and, increase clinicians’ awareness of the risk of hypoglycemia in their T2DM patients. This would contribute to improve the management of these patients and promote a therapeutic approach directed toward the patients’ needs, contributing to a better perception of HRQoL.

Phase 1 of the study resulted in the final version of the QoLHYPO^©^ questionnaire, consisting of a total of 13 items. The face validity and feasibility of the questionnaire showed that it is easy and quick to implement, proving its feasibility of use in routine clinical practice.

The evaluation of the psychometric properties of the questionnaire conducted during Phase 2 showed that the QoLHYPO^©^ questionnaire has a high degree of reproducibility and internal consistency, suggesting that, in identical situations, in which the patient’s clinical situation is the same but at different times, the results of the questionnaire would remain the same. The responsiveness analysis determined moderate internal responsiveness while no significant values were obtained in relation to external responsiveness. Finally, data regarding MCID showed that a 3.2 point difference in score on QoLHYPO^©^ between two administrations of the questionnaire was indicative of a change in patient health status, which was reflected in HRQoL. The use of the QoLHYPO^©^ questionnaire on the study population revealed the decline of T2DM patients’ HRQoL due to hypoglycemia. The aspects of HRQoL most affected by hypoglycemia were social relationships and emotional state. These results are in line with previous studies that highlighted the impact of hypoglycemia on patients socially, emotionally, financially as well as on their HRQoL [9–17].

The diabetic population included in the study is representative of the Spanish population with T2DM in that their characteristics are similar to those described in previous studies conducted in the field of healthcare in Spain [2].

Regarding the study methodology, the use of Rasch analysis to develop the questionnaire is worthy of note. The most common methodologies for the analysis of questionnaires are Item Response Theory (IRT) and Classical Test Theory (CTT) [58]. Rasch analysis, which is a type of IRT, presents a number of advantages over CTT [58–60]. These include specific objectivity, that is, the difference between two individuals in the measurement of a construct does not depend on the specific items that have been used for the measurement. Similarly, the difference between two items does not depend on the specific individuals who are used in the measurement. Another advantage of Rasch analysis is the conjoint measurement, which seeks to express parameters for people and items on the same measurement scale, allowing interaction between items and persons. Then there is the interval property, in other words, the interpretation of differences on the scale is the same throughout. This is a fundamental property for the analysis of change. And the last advantage is the use of infit and outfit statistics to identify the items and individuals that do not fit the model.

This study has certain limitations. Most of them are inherent to the methods used for the ICC analysis, the estimation of responsiveness and MCID. The main limitation related to ICC is its dependence on the variability of the sample used. Despite this limitation, none of the alternative methods are as objective as the estimation of ICC [38, 61]. In relation to the analysis of responsiveness, it is important to bear in mind that the literature does not provide a clear definition or a gold standard for its determination [43, 44]. Some authors distinguish between internal and external responsiveness [43], while others make a distinction between three categories, depending on the type of change that the questionnaire is able to detect [60]. Although this study uses the first classification, the assessment of responsiveness using other definitions and perspectives might provide new information. Lastly, following the recommendations found in the literature, anchor questions were used regarding the perspectives of patients and physicians in order to calculate MCID. Thus, it would be interesting to perform further analyses using multiple independent anchor questions and to analyze responsiveness in different population samples. As the results of responsiveness and MCID depend on the study sample and its inherent characteristics and hence there is no single score change value that can be extrapolated to every patient sample, these additional analyses would allow confirming the responsiveness and MCID obtained in this study [62].

## Conclusions

Use of the QoLHYPO^©^ questionnaire allows assessing the impact of hypoglycemic episodes on the T2DM patients’ HRQoL in routine clinical practice. This would contribute to improve the management of these patients and promote a therapeutic approach directed toward the patients’ needs, contributing to a better perception of HRQoL.

The assessment of the psychometric properties of the QoLHYPO^©^ questionnaire has shown that it is a tool with a high degree of reproducibility and internal consistency. Likewise, it has moderate ability to detect real variations in patient health status that can involve changes in their HRQoL.

Implementing the QoLHYPO^©^ questionnaire may provide useful information to identify patients who are at a higher risk of suffering a greater impact on their HRQoL due to hypoglycemia and to help in designing and adopting strategies that improve the management of these patients.

## Additional files

Additional file 1: Characteristic curves from first Rasch analysis. (DOCX 350 kb)

Additional file 2: Characteristic curves from second Rasch analysis (after recoding the response categories). (DOCX 314 kb)

Additional file 3: CUESTIONARIO QoLHYPO. (DOCX 24 kb)

## Abbreviations

ADDQoL: Audit of diabetes-dependent quality of life
AEMPS: *Agencia Española del medicamento y productos Sanitarios* (Spanish Agency for Medicines and Medical Devices)
AUC: Area under the curve
CTT: Classical test theory
DM: Diabetes mellitus
EQ-5D: EuroQoL-5d
HbA1c: Glycated Haemoglobin
HRQoL: Health-related quality of life
ICC: Intra-class correlation coefficient
IRT: Item response theory
MCID: Minimal clinically important difference
ROC: Receiver operating characteristic curves
SD: Standard deviation
SF-36: Short form 36
SRM: Standardized response mean
T2DM: Type-2 diabetes mellitus
VAS: Visual analog scale
