# Supplementary figures and images for: Impact of hypoglycemic episodes on health-related quality of life of type-2 diabetes mellitus patients: development and validation of a specific QoLHYPO© questionnaire
Source: Health Qual Life Outcomes. 2018 Mar 23;16:52. doi: 10.1186/s12955-018-0875-1 (PMC5865352; doi:10.1186/s12955-018-0875-1)

**Characteristic curves from first Rasch analysis**


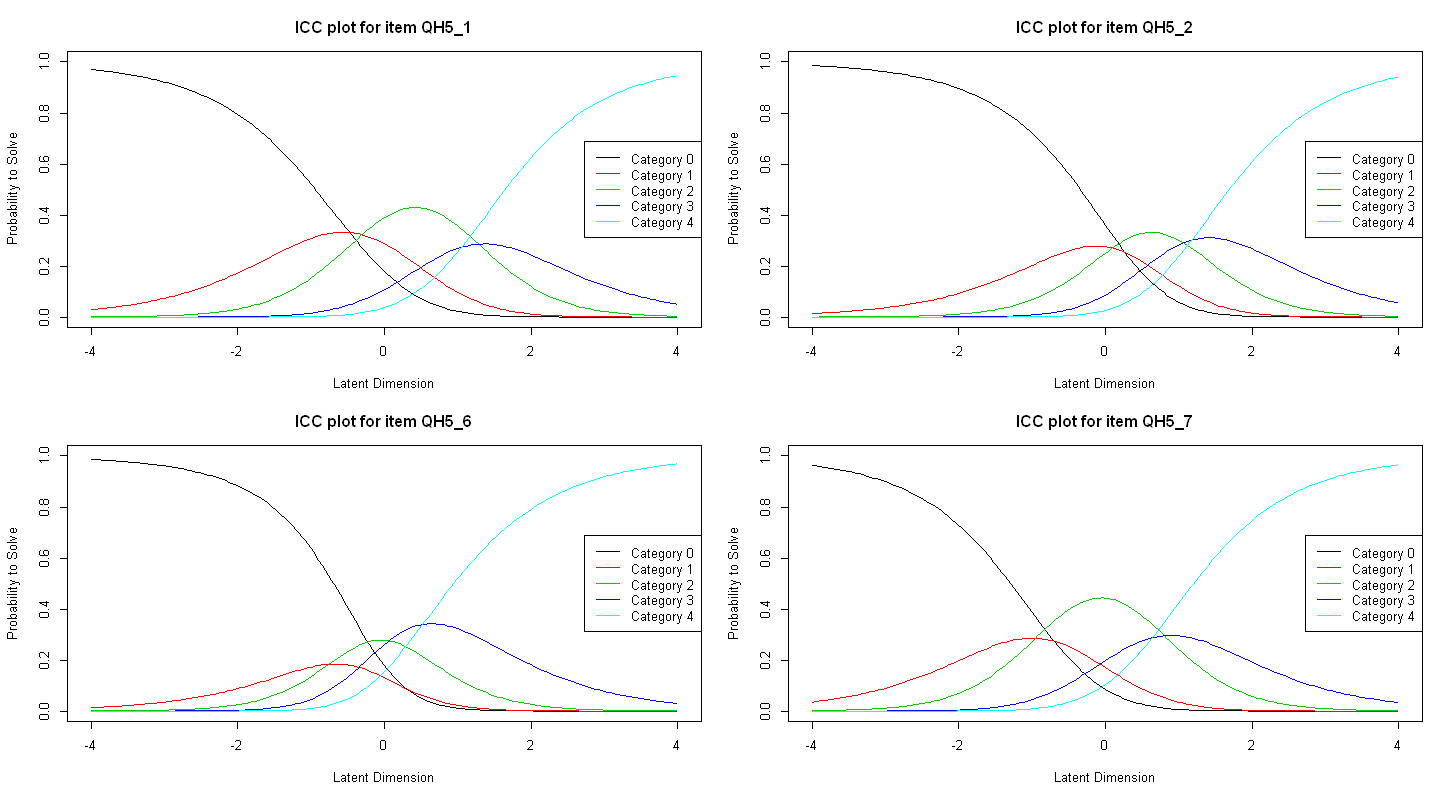

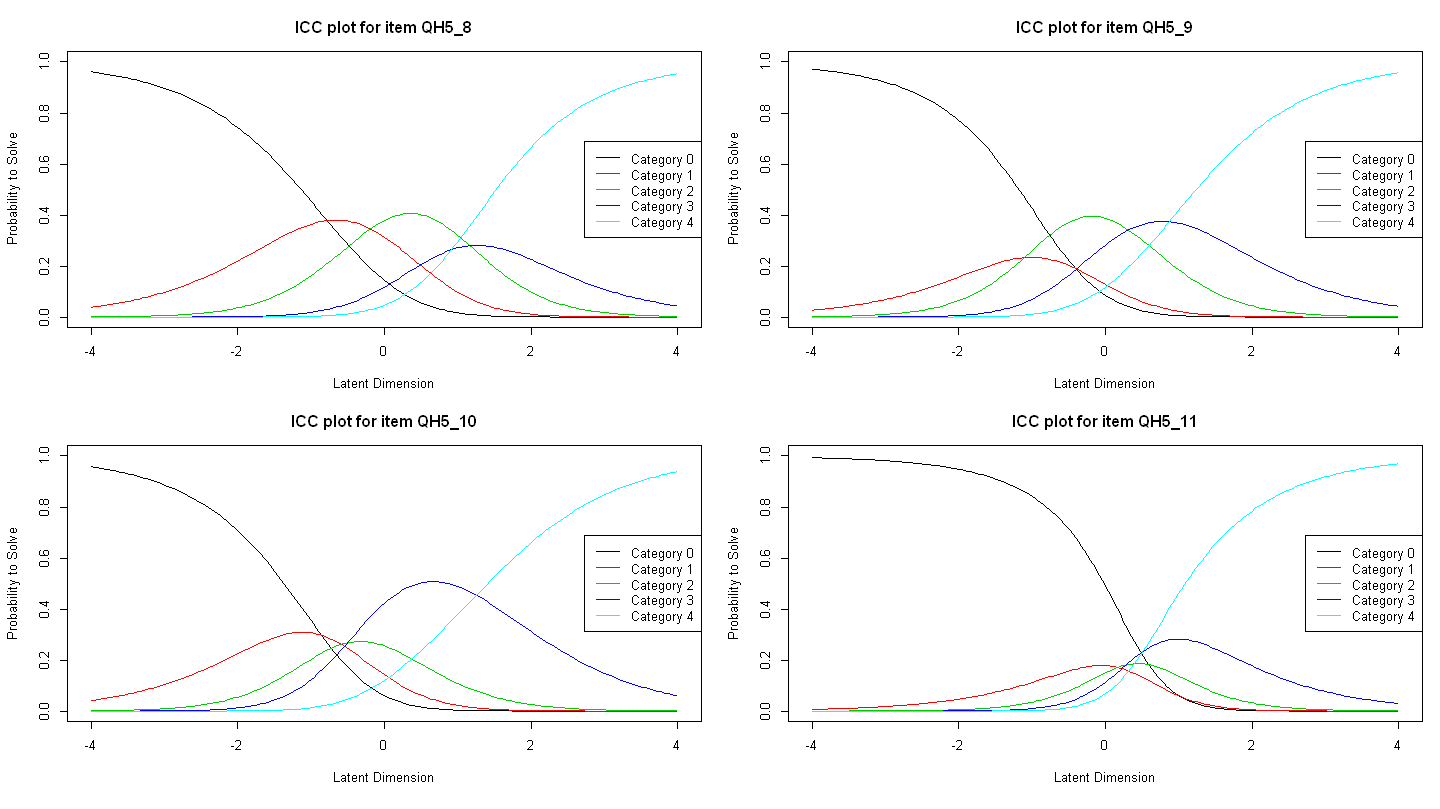

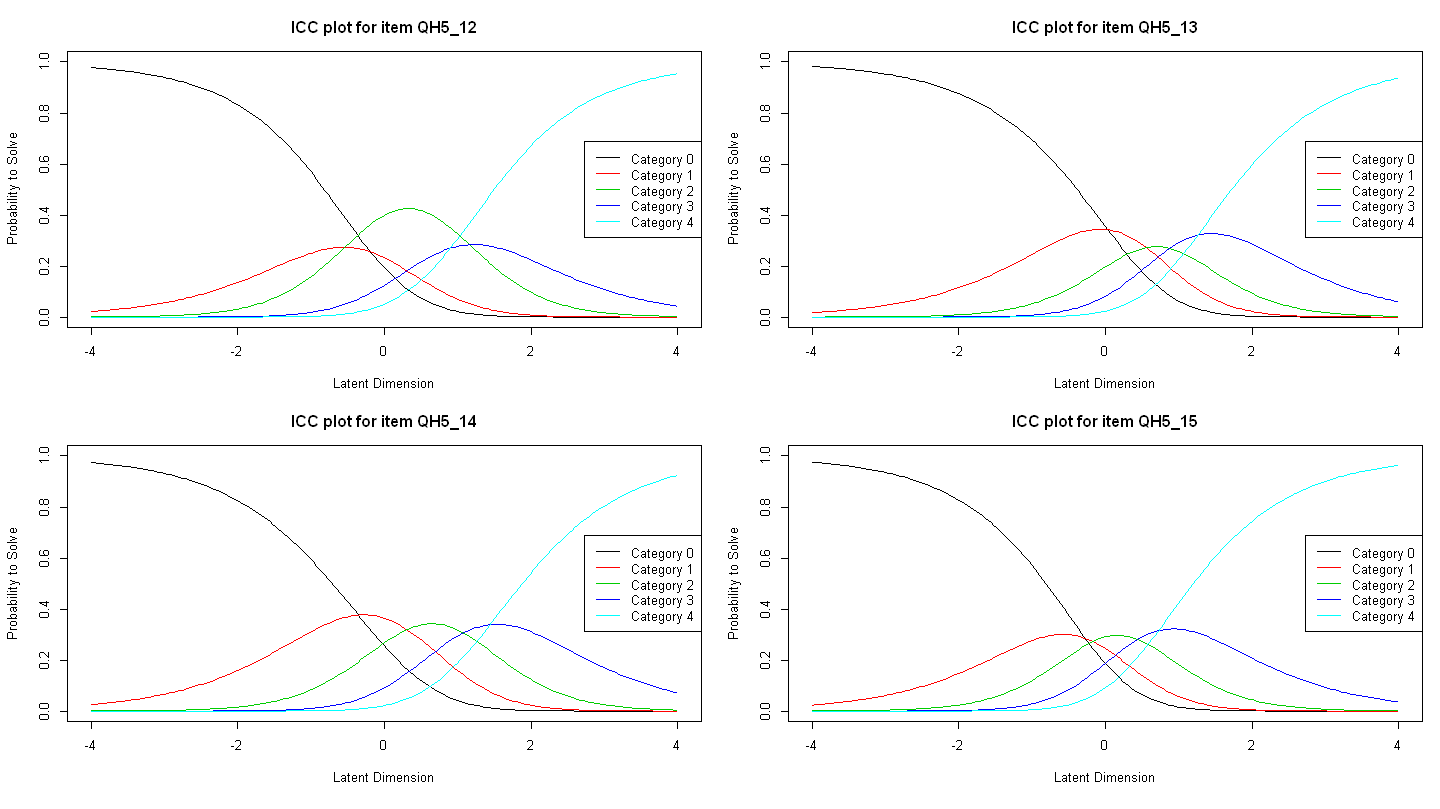

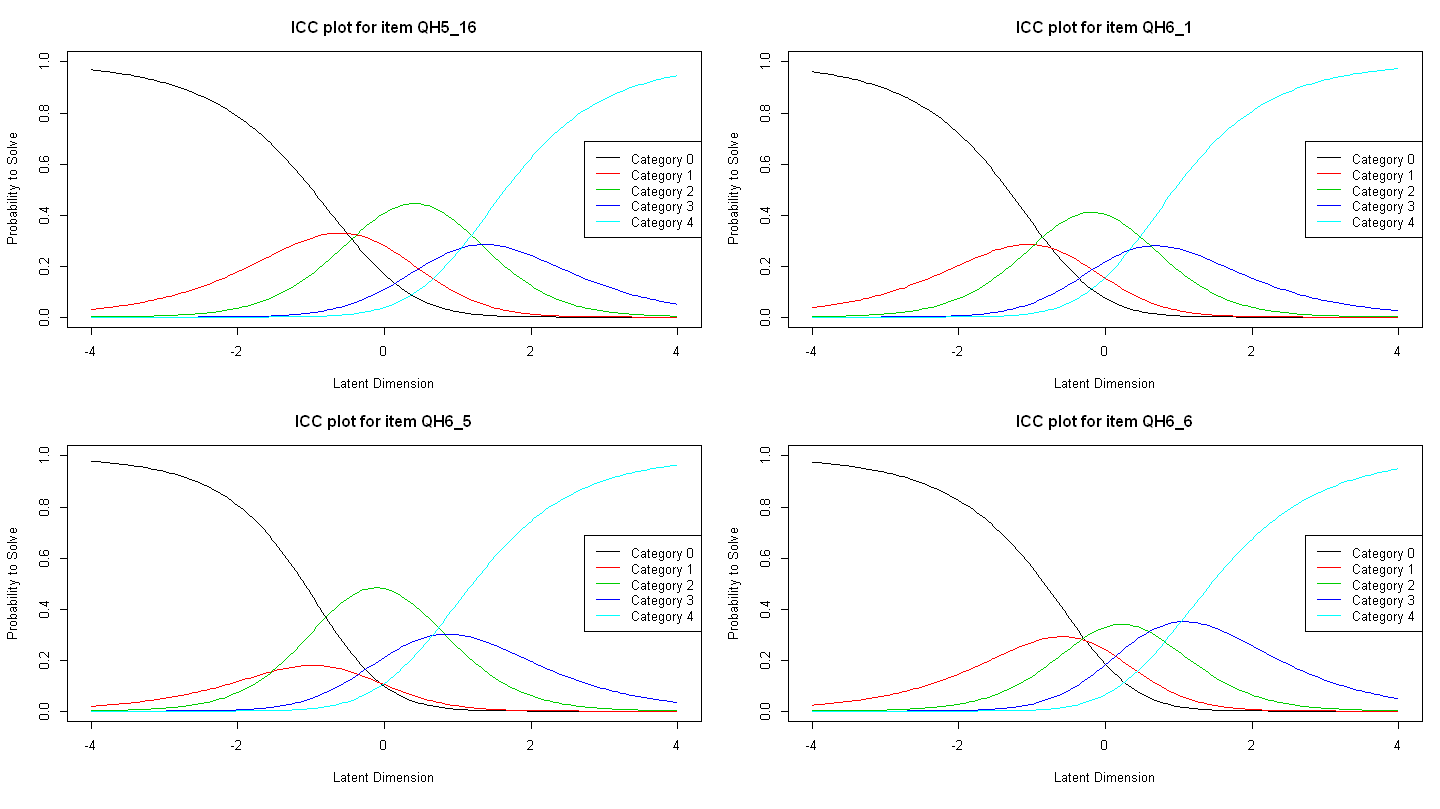

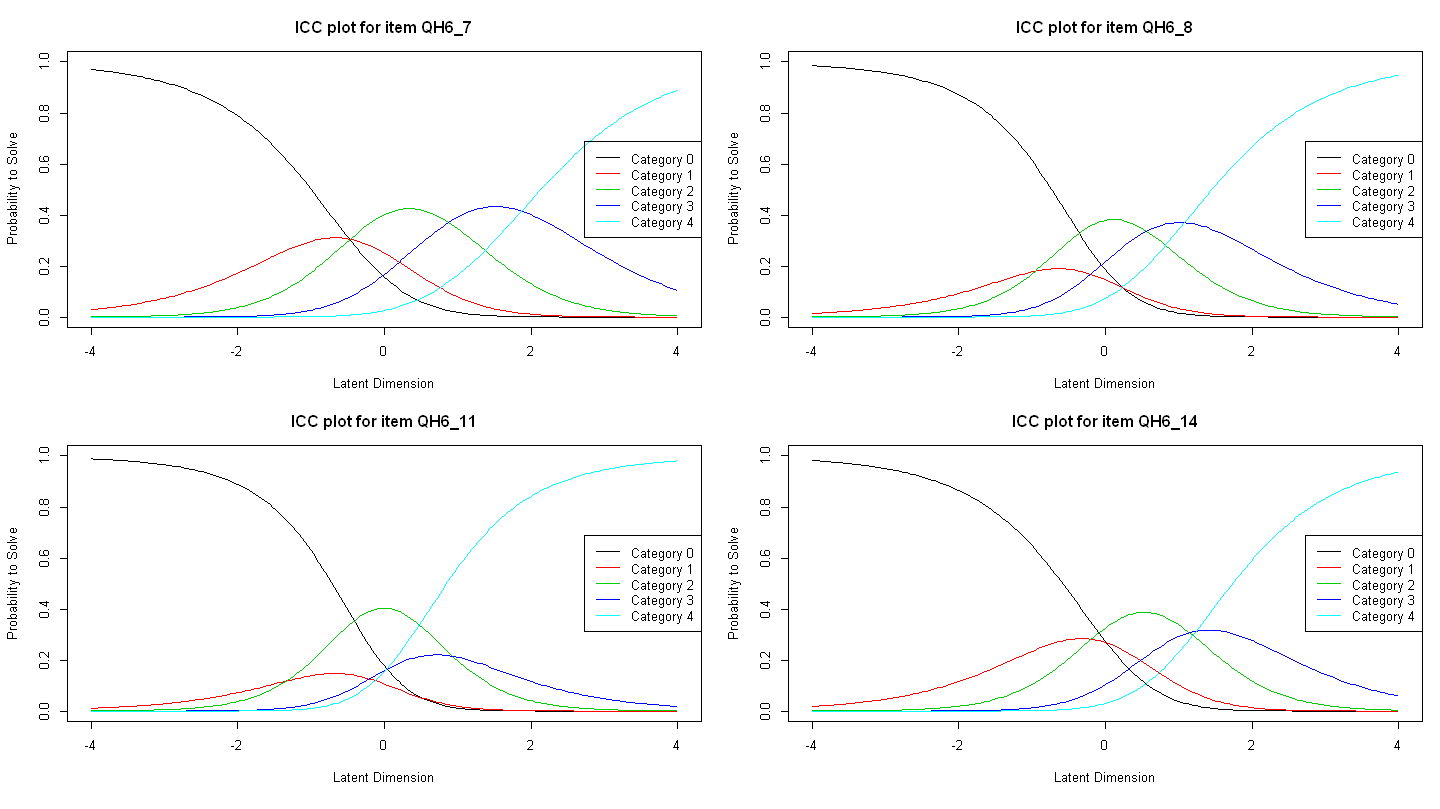

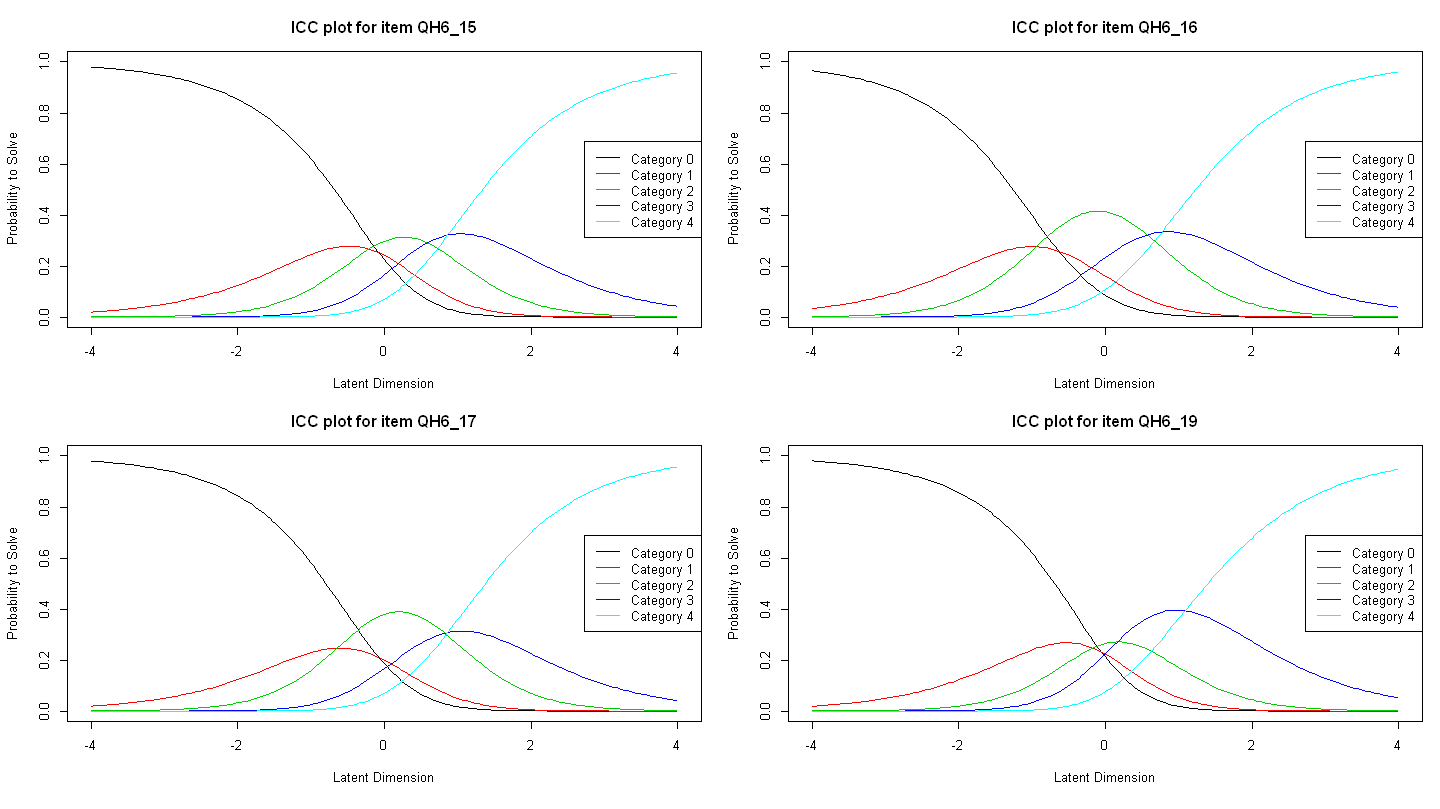

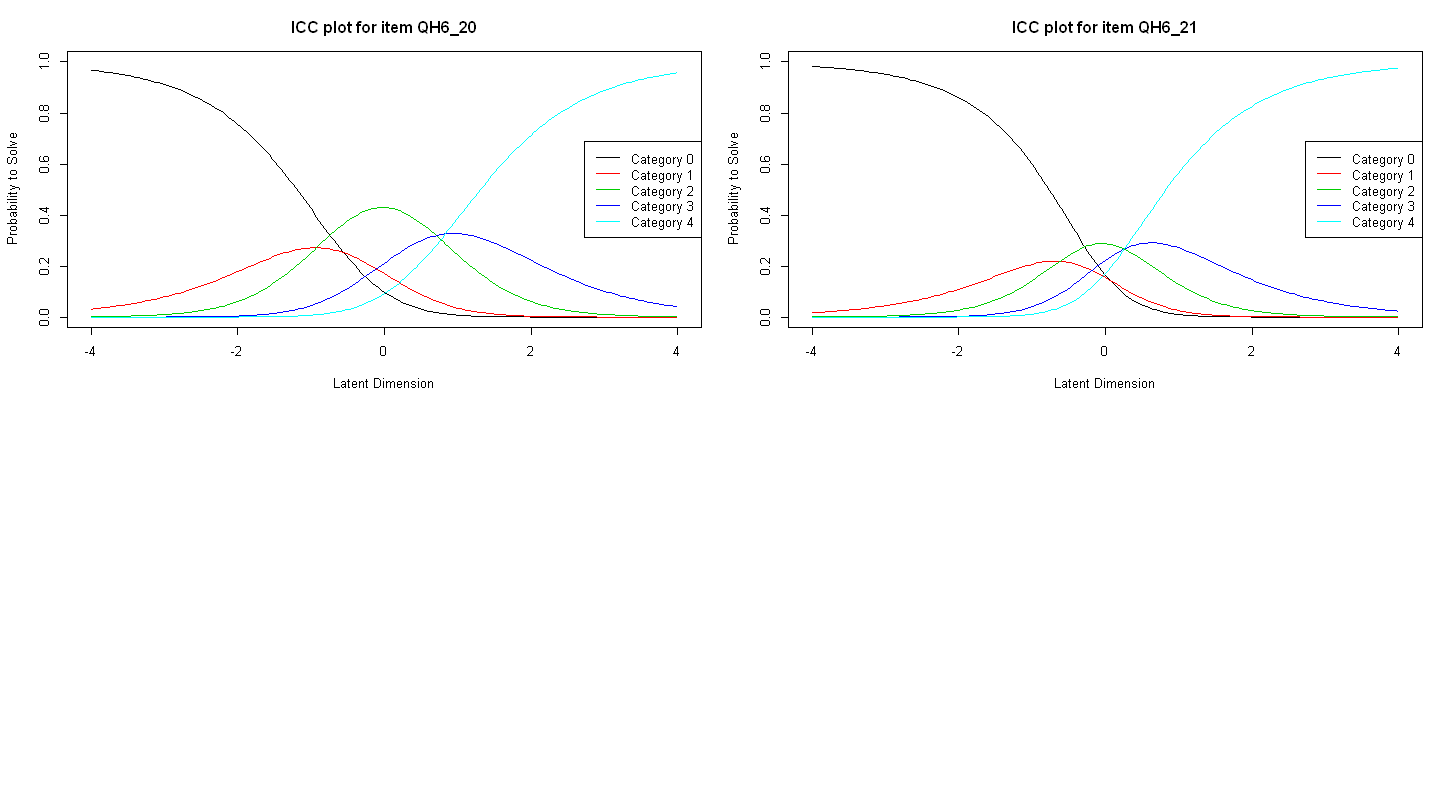

Supplement: Supplementary file 1 — Characteristic curves from first Rasch analysis. (DOCX 350 kb) [file 12955_2018_875_MOESM1_ESM.docx]

**Characteristic curves from second Rasch analysis (after recoding the response categories)**


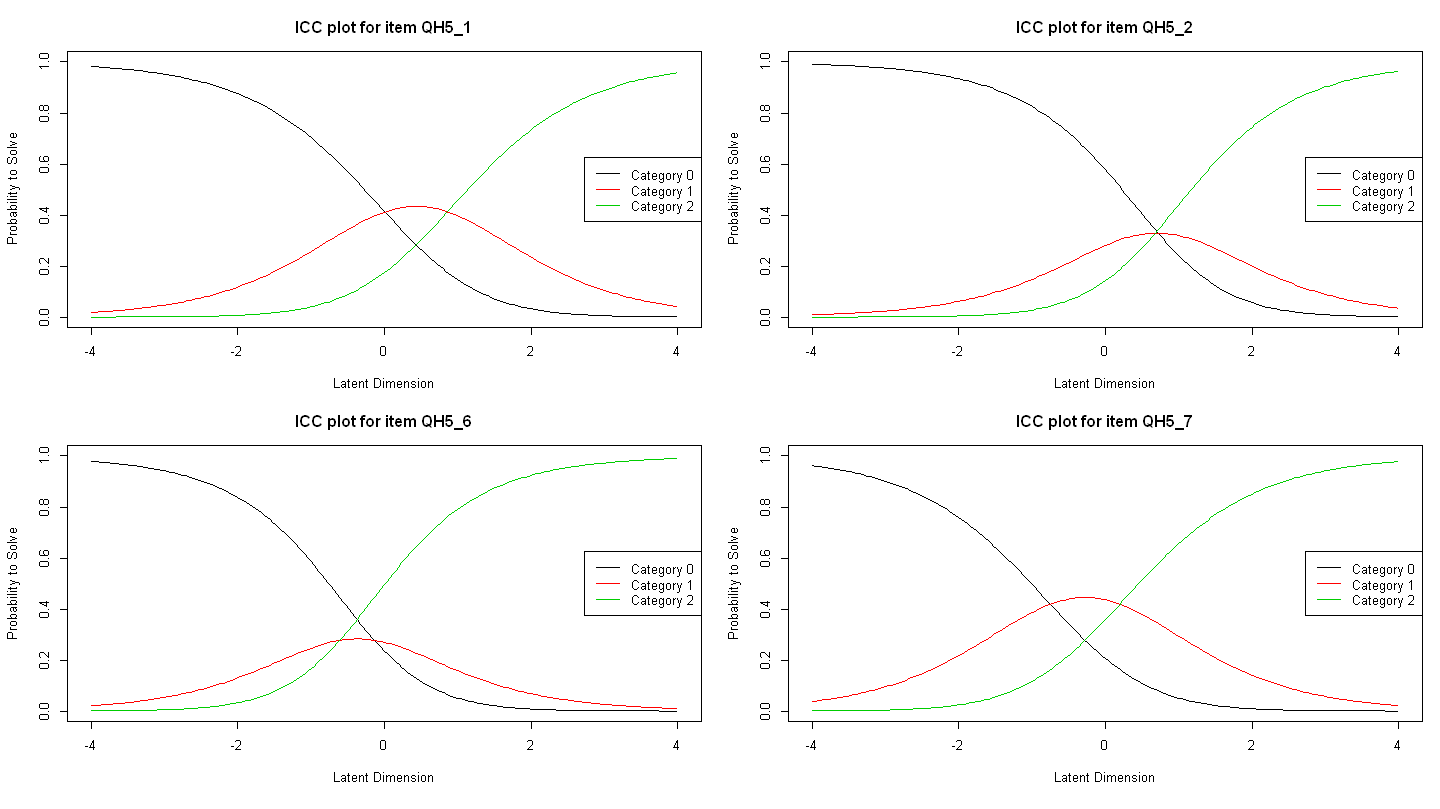

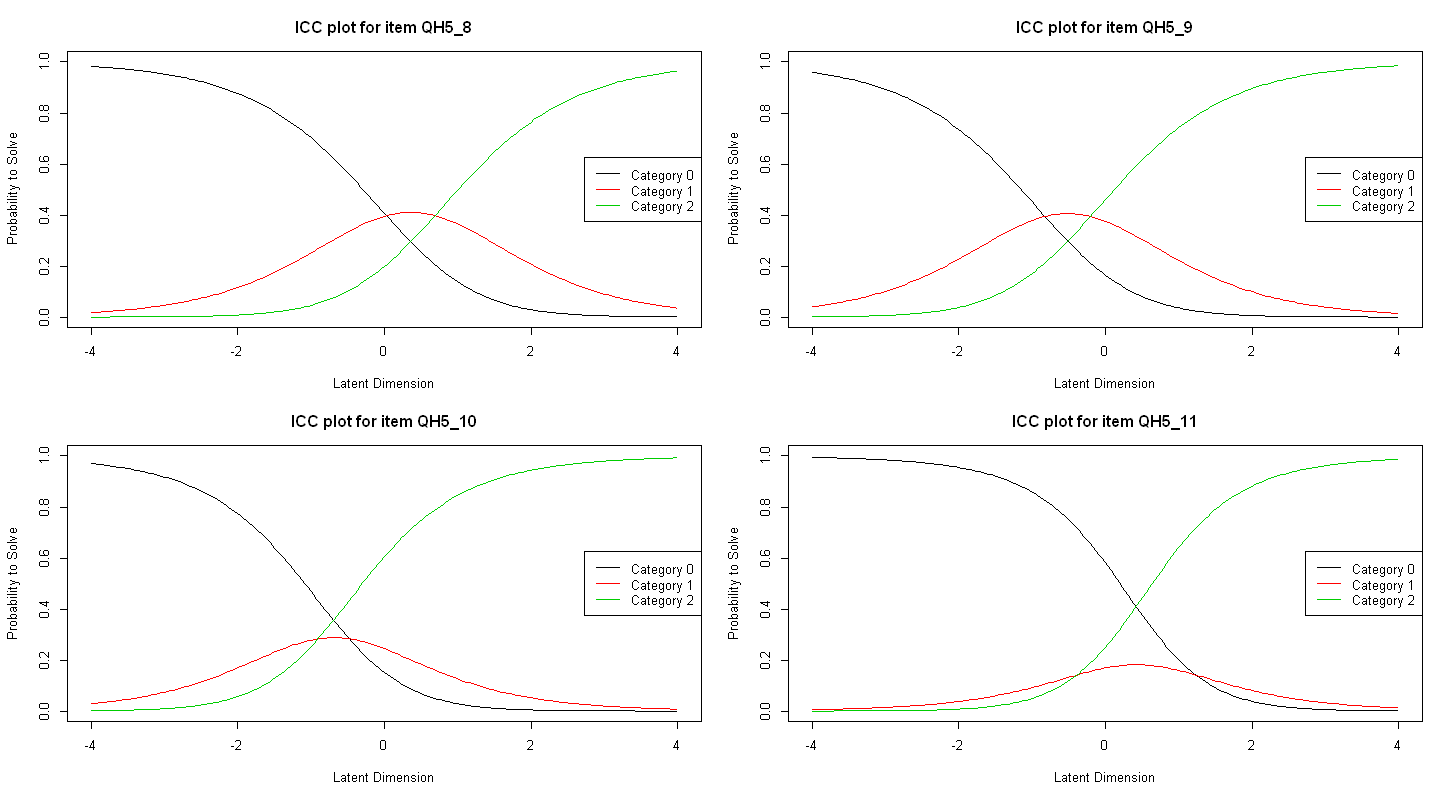

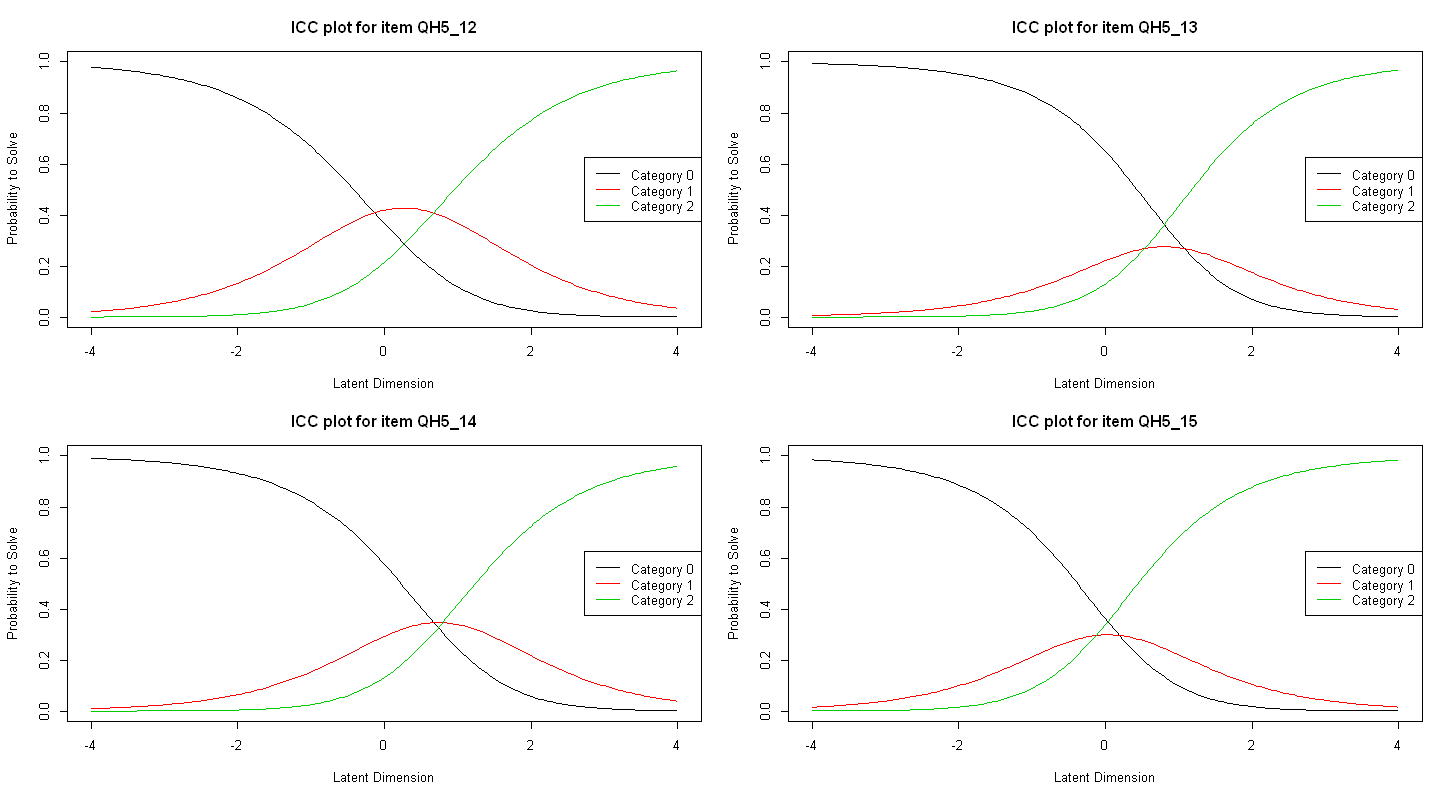

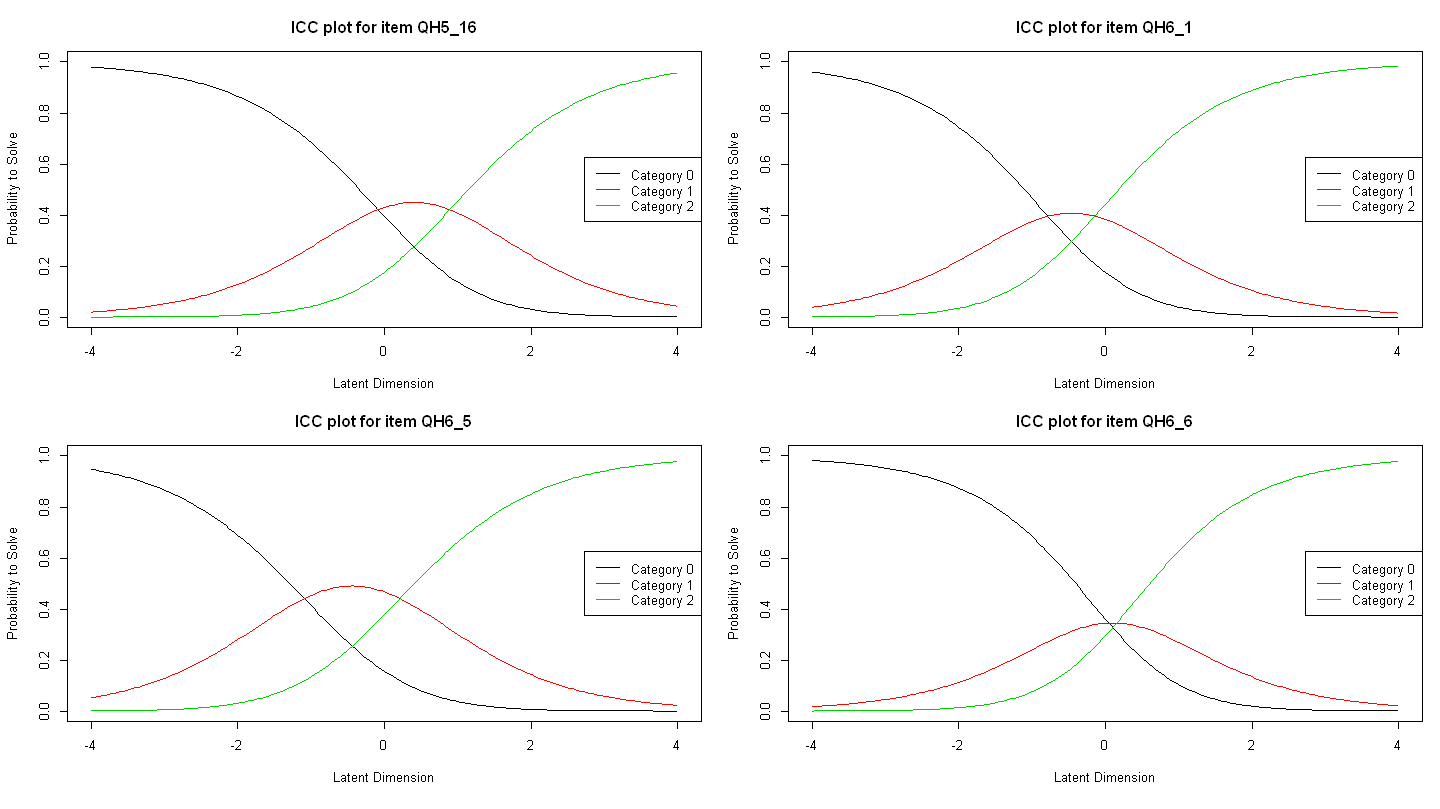

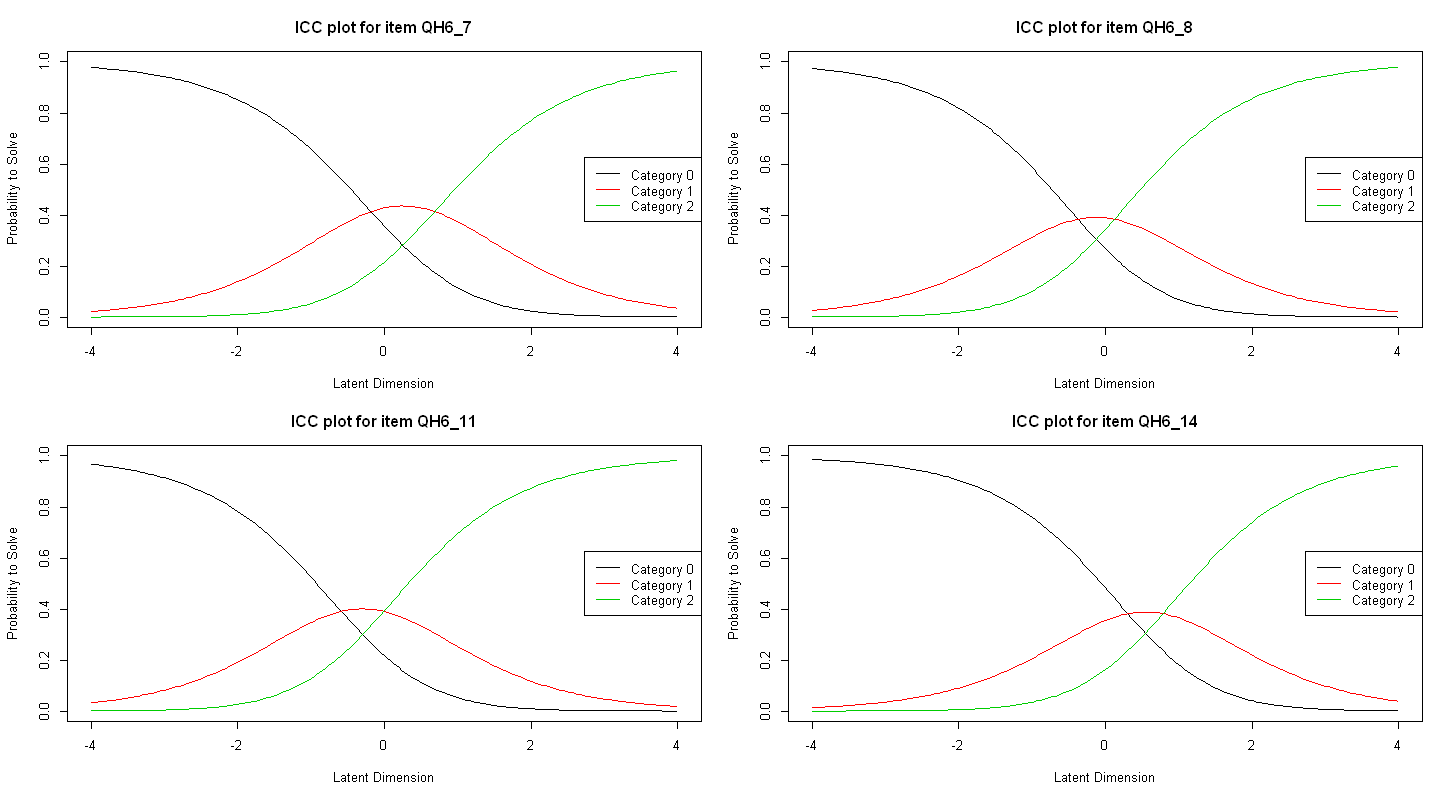

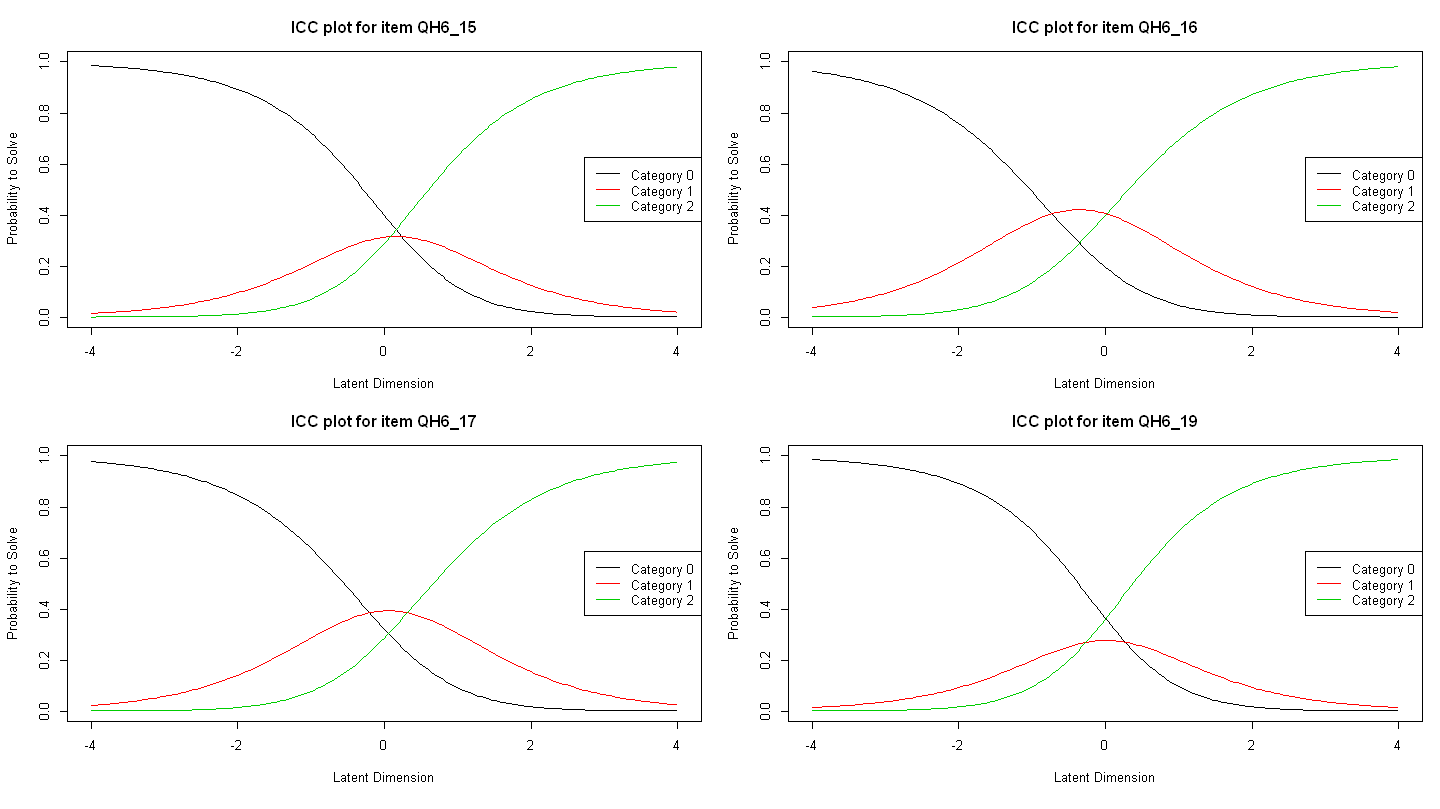

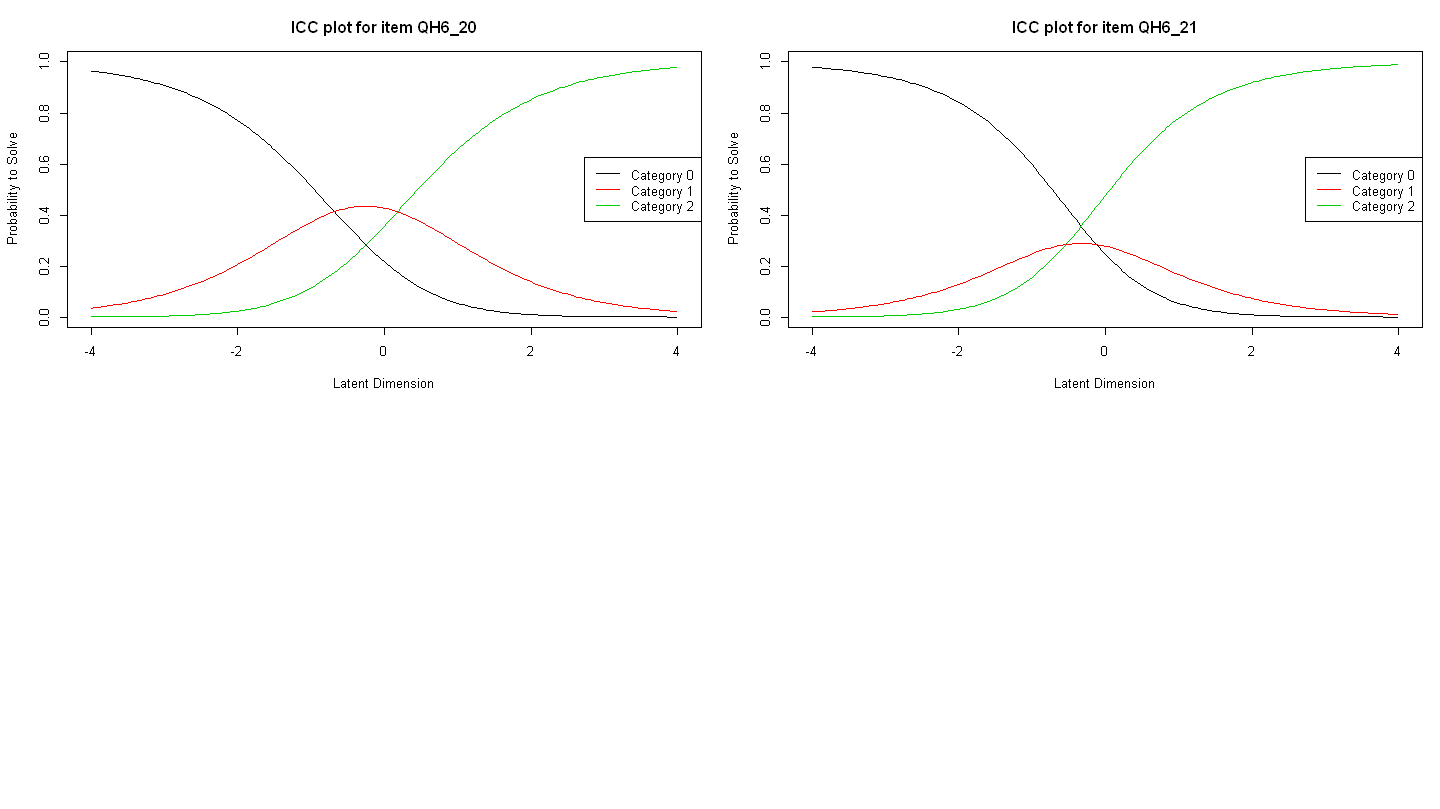

Supplement: Supplementary file 2 — Characteristic curves from second Rasch analysis (after recoding the response categories). (DOCX 314 kb) [file 12955_2018_875_MOESM2_ESM.docx]
