# Supplementary material for: Impact of hypoglycemic episodes on health-related quality of life of type-2 diabetes mellitus patients: development and validation of a specific QoLHYPO© questionnaire
Source: Health Qual Life Outcomes. 2018 Mar 23;16:52. doi: 10.1186/s12955-018-0875-1 (PMC5865352; doi:10.1186/s12955-018-0875-1)
Supplement: Supplementary file 3 — CUESTIONARIO QoLHYPO. (DOCX 24 kb) [file 12955_2018_875_MOESM3_ESM.docx]

| **CUESTIONARIO QoLHYPO** |
| --- |
| **A continuación le haremos una serie de preguntas para valorar cómo afectan sus hipoglucemias a su calidad de vida relacionada con su salud. Por hipoglucemia entendemos una bajada de azúcar.**   1. Señale con una X con qué frecuencia sufre bajadas de azúcar desde que fue diagnosticado de diabetes mellitus tipo 2:   Casi nunca  A veces  A menudo  Siempre   1. Señale con una X cómo valora usted la gravedad de la mayoría de sus bajadas de azúcar:   Muy leves  Leves  Moderadas  Graves  Muy graves   1. Señale con una X cómo valora usted sus conocimientos para controlar una bajada de azúcar:   No tengo conocimientos  Tengo conocimientos pero necesitaría más  Mis conocimientos son buenos  Mis conocimientos son muy buenos   1. Señale con una X con qué frecuencia utiliza el glucómetro para asegurarse de que está sufriendo una bajada de azúcar:   Nunca  Casi nunca  A veces  A menudo  Siempre  **5.** Marque con una cruz la opción de la parte derecha que mejor represente su respuesta y situación en cada caso. No deje ninguna cuestión sin marcar. Recuerde que no existen respuestas buenas ni malas.   \| **Cuando tengo una bajada de azúcar…** \| **Nunca** \| **A veces** \| **Siempre** \| \| --- \| --- \| --- \| --- \| \| se me pasan las ganas de hablar con la gente \|  \|  \|  \| \| estoy decaído, porque a pesar de seguir los consejos del profesional sanitario he tenido una bajada \|  \|  \|  \| \| me pongo de mal humor \|  \|  \|  \| \| me despierto en mitad de la noche y me cuesta dormirme las noches siguientes \|  \|  \|  \| \| me cuesta mucho hacer las tareas de casa \|  \|  \|  \| \| la sensación de cansancio hace que no tenga ganas de nada el resto del día \|  \|  \|  \|   **6.** A continuación, marque con una cruz la opción de la parte derecha que mejor represente su situación en cada una de las afirmaciones que se presentan. No deje ninguna cuestión sin marcar. Recuerde que no existen respuestas buenas ni malas.   \| **Piense en cada una de las siguientes frases en relación con sus bajadas de azúcar.** \| **Nunca** \| **A veces** \| **Siempre** \| \| --- \| --- \| --- \| --- \| \| Las bajadas de azúcar afectan la relación con mi familia \|  \|  \|  \| \| A causa de las bajadas de azúcar, pierdo confianza en mí mismo \|  \|  \|  \| \| En general, me siento más preocupado por si tengo una bajada de azúcar \|  \|  \|  \| \| Cuando me voy a dormir tengo miedo a que me dé una bajada de azúcar mientras duermo \|  \|  \|  \| \| Las bajadas de azúcar me impiden desarrollar mi trabajo con normalidad \|  \|  \|  \| \| Hago menos ejercicio físico del que debería por miedo a sufrir una bajada de azúcar \|  \|  \|  \| \| Tener que comer sin hambre para evitar las bajadas de azúcar, me resulta molesto \|  \|  \|  \| \| **©2017 Novartis Farmacéutica, S.A.U.** \| \| \| \| |
